# Post-translational modifications of the apelin receptor regulate its functional expression

**DOI:** 10.3934/Neuroscience.2023022

**Published:** 2023-10-31

**Authors:** Toshihiko Kinjo, Shun Ebisawa, Tatsuya Nokubo, Mifu Hashimoto, Takonori Yamada, Michiko Oshio, Ruka Nakamura, Kyosuke Uno, Nobuyuki Kuramoto

**Affiliations:** Laboratory of Molecular Pharmacology, Faculty of Pharmaceutical Sciences, Setsunan University, Hirakata, Osaka 573-0101, Japan

**Keywords:** apelin/apelin receptor system, neurobiology, post-translational modifications, functional expression, glycosylation, ubiquitination

## Abstract

Post-translational modifications (PTMs) are protein modifications that occur after protein biosynthesis, playing a crucial role in regulating protein function. They are involved in the functional expression of G-protein-coupled receptors (GPCRs), as well as intracellular and secretory protein signaling. Here, we aimed to investigate the PTMs of the apelin receptor (APLNR), a GPCR and their potential influence on the receptor's function. In an in vitro experiment using HEK cells, we only observed glycosylation as a PTM of the APLNR and ineffective receptor signaling by the agonist, (Pyr^1^)-apelin-13. In contrast, when analyzing mouse spinal cord, we detected glycosylation and other PTMs, excluding isopeptidation. This suggests that additional PTMs are involved in the functional expression of the APLNR in vitro. In summary, these findings suggest that the APLNR in vivo requires multiple PTMs for functional expression. To comprehensively understand the pharmacological effects of the APLNR, it is essential to establish an in vitro system that adequately replicates the receptor's PTM profile. Nonetheless, it is crucial to overcome the challenge of heat-sensitive proteolysis in APLNR studies. By elucidating the regulation of PTMs, further research has the potential to advance the analysis and pharmacological studies of both the apelin/APLNR system and GPCR signal modulation.

## 1. Introduction

The apelin receptor (APLNR) is a type of G-protein-coupled receptor (GPCR), which has garnered significant attention since its sequence similarity to the angiotensin (AT1) receptor was discovered in 1993 [1]. Apelin receptor, coupled with inhibitory G proteins, inhibits adenylate cyclase activity, counteracting the vasoconstrictive effects of angiotensin II and, consequently, modulating blood pressure [2]. Apelin, an APLNR agonist, was discovered in 1998 in bovine stomach extracts [3]. Since then, the intracellular signaling of the apelin/APLNR system has been well studied. This system stimulates signaling pathways, such as phosphoinositide 3-kinase (PI3K)/protein kinase B (Akt) cascades and mitogen-activated protein kinase (MAPK) cascades, which are involved in various cellular signaling pathways, including proliferation and cell death. Akt activation occurs through phosphorylation at serine 473 (S473) by the mammalian target of rapamycin complex 2 (mTORC2), followed by phosphorylation at threonine 308 (T308) by phosphoinositide-dependent protein kinase 1 (PDK1) [4]. Extracellular signal-regulated kinases (ERK)1/2 (p44/42) are members of MAPKs and are activated by upper kinases called MAP kinase kinases via phosphorylation at T202 and Tyrosine 204 (Y204) on ERK1 or T185 and Y187 on ERK2 [5]. In addition to its role in blood pressure regulation [6], the apelin/APLNR system is involved in various mechanisms, including angiogenesis [7], developmental normalization [8],[9] and neuroprotection [10]. Among these, we focused on the neuroprotective effects of the apelin/APLNR system. Cortical neurons transfected with APLNR exhibit neuroprotective properties against NMDA excitotoxicity by activating PI3K/Akt and Raf/ERK cascades [11]. Furthermore, in vivo studies involving tissues such as the mouse hippocampus [11] and retina [12] have demonstrated the neuroprotective effects of APLNR activation in vivo. Pretreatment with apelin reduces neuronal death in the ischemic mouse brain [13]. Evidence suggests that this system may be a potential therapeutic target for neurodegenerative diseases. In our previous study, Apelin knockout mice exhibited accelerated disease progression in amyotrophic lateral sclerosis (ALS) model, whereas increased survival of APLNR-expressing motor neurons was observed in ALS mice [13], suggesting that the APLNR is involved in the maintenance of motor neuron survival. Although the function of the APLNR has been elucidated, its wide range of effective concentrations remain a pharmacological enigma [14],[15]. The factors contributing to this require clarification prior to the use of APLNR as a therapeutic target.

Post-translational modifications (PTMs) are responsible for the activation or termination of protein function [16]. More than 500 types of PTMs have been reported and registered in the unimod database (http://www.unimod.org/). Among them, phosphorylation is a well-known PTM that is essential for the activation or termination of enzymatic reactions and signal transduction. Glycosylation profoundly affects protein conformation, including folding, localization and stability. Ubiquitination of lysine residues promotes proteasomal degradation, whereas the addition of a small ubiquitin-related modifier (SUMO, i.e., SUMOylation) to lysine residues protects against degradation [17]. The function, localization and stability of GPCRs are also affected by PTMs, including phosphorylation, glycosylation, nitrosylation, tyrosine sulfation, palmitoylation, ubiquitination and SUMOylation [19]. Phosphorylation and glycosylation of the APLNR have been reported and predicted PTMs on the APLNR include SUMOylation and palmitoylation [20]. Although PTMs in most receptors can be inferred from databases and in silico approaches, the precise mechanisms underlying these modifications remain to be elucidated. The addition of functional groups, such as phosphorylation and palmitoylation does not significantly change the molecular weight of proteins, whereas isopeptidation, including ubiquitination and SUMOylation, or glycosylation with a polysaccharide chain, inevitably causes distinct changes in molecular weight. In this study, we investigated the precise signaling mechanism of the APLNR by overexpressing it and explored the potential PTMs involved. We seek to gain a comprehensive understanding of the pharmacological properties of these receptors. We specifically addressed the possibility that certain PTMs of the APLNR, which were not detected in vitro, may stabilize this protein and contribute to its function in vivo.

## 2. Materials and methods

### 2.1. Materials

(Pyr^1^)-Apelin-13 was purchased from Peptide Institute (Osaka, Japan). Dulbecco's modified Eagle's medium (DMEM), Blocking One Histo and FluoroKEEPER antifade reagent were purchased from Nacalai Tesque (Kyoto, Japan). ISOGEN II RNA extraction reagent (Nippon Gene, Tokyo, Japan) and ReverTra Ace qPCR RT master mix were provided by Toyobo (Osaka, Japan). Forskolin was supplied by Merk-Sigma (Burlington, MA); polyvinylidene difluoride (PVDF) membrane was obtained from Merk-Millipore (Burlington, MA); Western Lightning Plus-ECL was purchased from PerkinElmer (Waltham, MA); protein A-sepharose 4B resin and EZ-Link NHS-LC-Biotin and Pierce NeutrAvidin UltraLink Resin were purchased from Thermo Scientific (Waltham, MA). PNGase F was provided by New England BioLabs (Ipswich, MA); USP2, Den1 and SENP1 were purchased from LifeSensors (Malvern, PA). All other chemicals used were of the highest commercially available purity.

### 2.2. Antibodies

Rabbit IgG anti-phospho-PKA substrate (RRXS*/T*) (100G7E), 1:1000 (#9621); anti-Akt (pan), 1:2000 (#3673); anti-phospho-Akt (T308), 1:1000 (#2965); phospho-Akt (S473), 1:2000 (#4060); anti-ERK1/2, 1:2000 (#4695); phospho-ERK1/2 (Thr202/Tyr204), 1:2000 (#4370); Alexa Fluor® 555-conjugated goat IgG anti-mouse IgG (H+L) and F(ab')2 Fragment were purchased from Cell Signaling Technology (Danvers, MA). Mouse IgG anti-human APJ (APLNR) was obtained from R&D systems (Minneapolis, MN), 1:1000 (#MAB8561). Mouse IgG anti-GAPDH, 1:10000 (#014-25524) and anti-FLAG were from FUJIFILM Wako Pure Chemical Corporation (Osaka, Japan), I.F. 1:200, W.B. 1:1000 I.P. 1:500 (#014-22383). Rabbit IgG anti-myelin protein zero was from Abcam (Cambridge, UK), 1:1000 (#ab31851).

### 2.3. Animals

All procedures were performed according to the guidelines of the Japanese Society for Pharmacology and approved by the Committee for Ethical Use of Experimental Animals at Setsunan University. Adult (5–6 weeks old) male C57BL/6 mice were housed at our facilities under a light–dark cycle of 12–12 hr and a humidity of 55% at 23 °C and provided with ad libitum access to food and water.

### 2.4. Cell culture

HEK293TN cells (System Bioscience, CA, USA) were cultured in DMEM containing 10% fetal bovine serum and 1% penicillin-streptomycin at 37 °C with 5% CO_2_. Cells reached about 80% confluency and were removed from the plate by Trypsin/EDTA and re-seeded at a density of 1.0 × 10^5^ cells/ml. Under these conditions, cells needed three days to reach about 80% confluency. During the culture period, drugs were added directly to the culture medium. Cells were exposed to (Pyr^1^)-apelin-13 for 30 min. In some cases, 15 min after the initiation of (Pyr^1^)-apelin-13 exposure, FSK exposure was performed for 15 min. Cells were exposed to tunicamycin at 1 day in vitro (DIV) for 48 h.

### 2.5. Plasmid

pcDNA3.1^+^-DYK-human APLNR (OHu11900) expressing the human APLNR protein with an N-terminal DYKDDDDK (FLAG) tag in mammalian cells (pcDNA-APLNR) was purchased from GenScript Biotech Corporation (Piscataway, NJ). An empty vector, pCDH-CMV-MCS-EF1-GreenPuro (CD513B-1; pCDH-GFP), was purchased from System Bioscience (CA, USA), and the FLAG-tagged APLNR gene from the abovementioned plasmid was subcloned (pCDH-GFP-APLNR). Mammalian cells transfected with this plasmid express APLNR and GFP under the control of the CMV and EF1 promoters, respectively.

### 2.6. Transfection

The calcium phosphate transfection method was performed by mixing 1.0 µg of a plasmid containing HEPES-buffered saline and CaCl_2_ at a final concentration of 86 mM. The DNA mixture was allowed to stand at room temperature for 20 min and then added to the cell culture in a 35 mm dish with 2.0 mL of culture media. Subsequently, the cells were placed in a CO_2_ incubator for 2 days. For mock transfections, an equal volume of sterile purified water was used instead of the plasmid.

### 2.7. RT-PCR

Total RNA extracted using the ISOGEN II was treated at 65 °C for 5 min and cooled on ice. 1 µg of RNA as template was reacted with reverse transcription using ReverTra Ace qPCR RT master mix to prepare cDNA through at 37 °C for 15 min and 98 °C for 5 min. Gene transcripts were amplified using specific primer sets, as listed below.

**Table:** 

*APLNR* (207 bp)	Forward:	5′-TTCTGCAAGCTCAGCAGCT-3′
	Reverse:	5′-GGTGCGTAACACCATGACAG-3′
*GAPDH* (193 bp)	Forward:	5′-TCATTGACCTCAACTACATGGTCTA-3′
	Reverse:	5′-ACACCAGTAGACTCCACGACATACT-3′

Briefly, RT-PCR was performed in three steps. The first step consisted of 1 cycle at 94 °C for 120 s. The second step included 30 cycles at 94 °C for 45 s, 55 °C for 45 s and 72 °C for 30 s. The third step consisted of 1 cycle at 72 °C for 60 s. The PCR products were separated by electrophoresis using 0.8% of TAE agarose gel and visualized using the ImageQuant 400 (GE electronics, Boston, MA).

### 2.8. Immunoblotting

Immunoblotting was performed as previously described [21]. Cells were lysed using a buffer composed of 10 mM Tris-HCl (pH 7.5), 0.32 M sucrose, 1 mM EDTA, 1 mM EGTA, 5 mM dithiothreitol, phosphatase inhibitors [10 mM sodium b-glycerophosphate and 10 mM sodium pyrophosphate (PPA), 50 mM sodium fluoride (NaF)] and 1 µg/mL each of protease inhibitors ([p-amidinophenyl] methanesulfonyl ﬂuoride, benzamidine, leupeptin and antipain). In some cases, protease inhibitors were omitted. Proteins in the lysate were solubilized at 100 °C for 10 min, 37 °C for 10, 30 or 60 min or 4 °C for 10 min in a buffer containing 2% sodium dodecyl sulfate (SDS), 5% 2-mercaptoethanol, 10% glycerol and 0.01% bromophenol blue. Unless otherwise specified in the method, the solubilization was performed at 100 °C. Subsequently, 10 µg of proteins were subjected to 10% SDS–polyacrylamide gel electrophoresis and transferred onto PVDF membranes. To block non-specific binding, the membranes were immersed in Tris-buffered saline (TBS) containing 5% skim milk and 0.05% Tween-20 for 1 hr, followed by incubation with a specific primary antibody for 2 hr and with horseradish peroxidase-conjugated secondary antibody for 1 hr at room temperature. Membranes were subjected to detection using Western Lightning Plus-ECL and exposed to X-ray films (Fujifilm, Tokyo, Japan) for visualization. After detecting total protein, the membrane for detecting phosphorylation was soaked in WB stripping solution (Nacalai # 05364-55), gently shaken at room temperature for 30 minutes and used again for antibody reaction.

### 2.9. Immunocytochemistry

Cells on a coverslip were fixed with 4% paraformaldehyde in TBS for 10 min. To enhance membrane permeability, cells were treated with TBS containing 0.1% Triton X-100 for 10 min. After blocking with Blocking One Histo for 10 min, a specific primary antibody was applied in TBS with 0.1% Blocking One and 0.03% Tween-20 and incubated overnight at 4 °C. Cells were then incubated for 2 hr with appropriate combinations of secondary antibodies. After an additional wash with TBS, a coverslip containing immunostained cells was mounted using FluoroKEEPER antifade reagent. Immunostaining images were observed under a confocal laser-scanning microscope (LSM 510; Carl Zeiss, Jena, Germany).

### 2.10. Deglycosylation and deisopeptidation

PNGase F, USP2, Den1 and SENP1 were used to remove N-linked glycosylation, ubiquitination, NEDDylation or SUMOylation, respectively, according to the manufacturer's instructions. Briefly, when the protein required denaturing before reacting with the enzyme, 10 µg of protein was mixed with denaturing buffer containing 0.5% SDS and 40 mM DTT and incubated at 100 °C for 10 min. Subsequently, 10 µg of proteins were mixed with 50 mM PBS containing 1% NP-40 and incubated with 500 units/10 nmol PNGase F, 35 mmol USP2, 1 µmol Den1 or 10 nmol SENP1 at 37 °C for 1 h. The proteins from HEK293TN cells or the mouse spinal cord were solubilized at 37 °C or 100 °C for 10 min respectively and subjected to immunoblotting. In some cases, 100 µg/mL each of protease inhibitors ([p-amidinophenyl]methanesulfonyl ﬂuoride, benzamidine, leupeptin and antipain) were added to the reaction mixture.

### 2.11. Immunoprecipitation

Cells were lysed in RIPA buffer composed of 50 mM Tris-HCl (pH 8.0), 5 mM EGTA, 5 mM EDTA, 50 mM NaF, 10 mM PPA, 1% nonidet P-40 (NP-40), 0.5% deoxycholate and 0.1% SDS. The lysate containing 100 µg of proteins was incubated with 1 µg of anti-FLAG antibody and protein A-sepharose 4B resin by rotation mixer at 4 °C for 120 min. The bound substances were precipitated by centrifugation, and the precipitant was solubilized at 37 °C for 10 min and subjected to immunoblotting.

### 2.12. Membrane protein biotinylation

Cells were incubated with EZ-Link NHS-LC-Biotin at 4 °C for 60 min and lysed in RIPA buffer. Subsequently, 250 µg of the biotinylated membrane proteins were incubated with Pierce NeutrAvidin UltraLink Resin by rotation mixer at 4 °C for 120 min. The bound substances were precipitated by centrifugation, and the precipitant was solubilized at 37 °C for 10 min and subjected to immunoblotting.

### 2.13. Statistical analysis

Densitometric analysis of immunoreactive bands was performed using ImageJ (National Institutes of Health). All data were expressed as means ± SE, and statistical significance was determined using Bonferroni's test.

## 3. Results

### 3.1. The function of the APLNR is not observed by forced expression in cells

**Figure 1.**
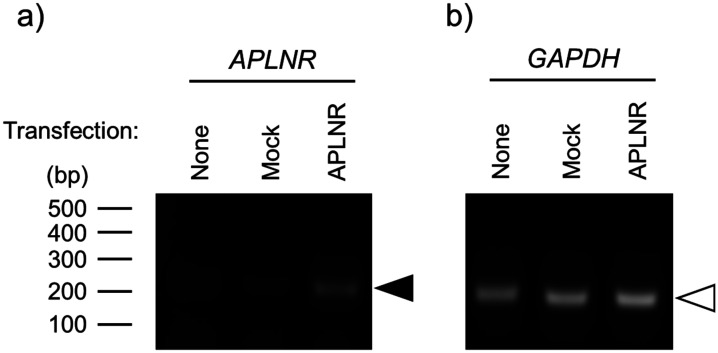
Overexpression of human APLNR in HEK293TN cells. Cells were cultured without any treatment (none) or transfected with a control (mock) or APLNR expression plasmid, pcDNA3.1-APLNR (APLNR), immediately after plating. After 3 days of transfection, cDNA from each condition was prepared, and a) APLNR or b) GAPDH gene expression was examined by PCR. Black and white arrowheads indicate the desired base pair of APLNR or GAPDH.

**Figure 2.**
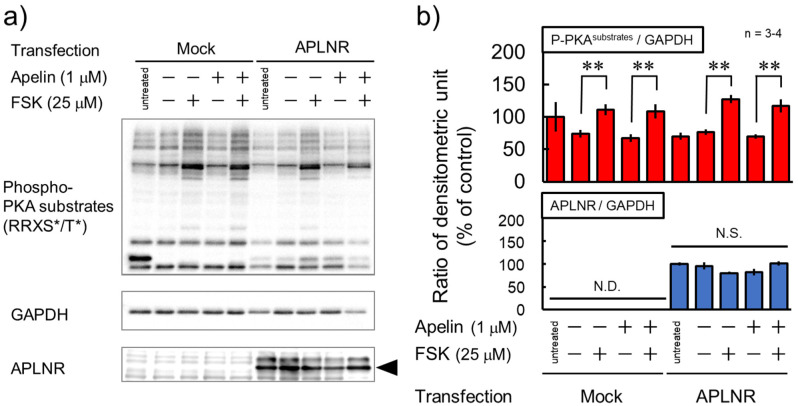
No significant alteration in phosphorylation levels of PKA substrates following apelin treatment. Cells transfected with a control (mock) or APLNR expression plasmid, pcDNA3.1-APLNR (APLNR), were collected after treatment with or without 1 µM (Pyr^1^)-apelin-13 for 30 min and/or 25 µM FSK for the last 15 min. a) Cell lysates were subjected to immunoblotting to detect the phosphorylated PKA substrates, GAPDH and APLNR. The black arrowhead indicates the desired molecular weight of the APLNR (42.6 kDa). b) In each lane of a), all band intensities for the phosphorylated PKA substrates or each band intensities for APLNR and GAPDH were quantified, and the ratio of densitometric units (phosphorylated PKA substrates or APLNR/GAPDH) was calculated. The “untreated” group is a sample that has not been treated with any drug, and the “negative (−)” group is a negative control that was added a solvent of drugs. The experiment was repeated three to four times. N.D. non-detectable. N.S. not significant. **P < 0.01 indicates significant differences between groups without FSK and with FSK (Bonferroni's test).

**Figure 3.**
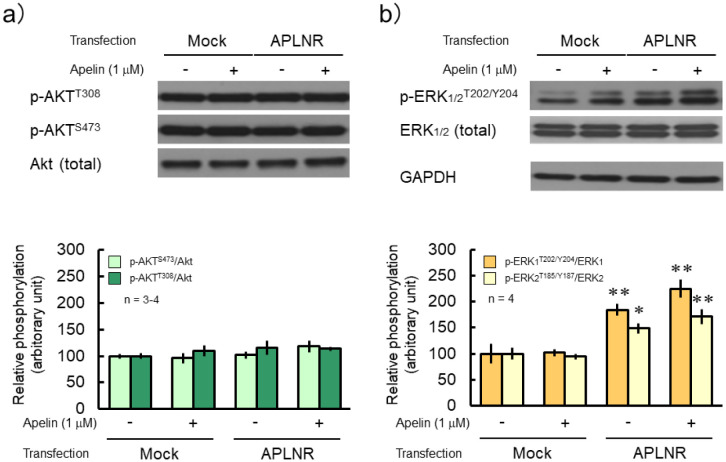
No significant alteration in total expression and phosphorylation levels of Akt or ERK following apelin treatment. Cells transfected with a control (mock) or APLNR expression plasmid, pcDNA3.1-APLNR (APLNR), were collected after treatment with or without 1 µM (Pyr^1^)-apelin-13 for 30 min. Cell lysates were subjected to immunoblotting to detect the total expression and phosphorylation levels of a) Akt or b) ERK_1/2_. GAPDH was used as an internal control. The experiment was repeated three or four times. The relative phosphorylation level was calculated by dividing the band intensity of each phosphorylation level by the total protein expression. *P < 0.05 and **P < 0.01 indicate significant differences between mock and APLNR transfections for each corresponding condition (Bonferroni's test).

The *APLNR* gene expression was observed when the expression vector was transfected into HEK293TN cells, while GAPDH gene expression was observed under all conditions (Fig. 1a, b). Forskolin (FSK) significantly increased the phosphorylation levels of several protein kinase A (PKA) substrates in both mock and APLNR-expressing cells, whereas (Pyr^1^)-apelin-13 did not affect phosphorylation levels (Fig. 2a). Furthermore, to investigate the expression of APLNR whether there was a change in its expression with treatment (Pyr^1^)-apelin-13 or FSK, the results showed that (Pyr^1^)-apelin-13 and FSK had no effect on APLNR expression, suggesting no membrane expression (Fig. 2b). GPCR-mediated activation of ERK has been shown to be facilitated by G protein-coupled receptor kinase (GRK) and β-arrestin, which are involved in the GPCR biased pathway [14]. In cortical neurons, APLNR activation stimulates PI3K/Akt and Raf/ERK cascades [11]. Relative phosphorylation levels of ERK_1/2_ were significantly higher in APLNR gene-transfected cells than in mock cells. However, (Pyr^1^)-apelin-13 did not alter the protein expression and phosphorylation levels of Akt and ERK_1/2_ (Fig. 3). These results suggest that APLNR protein expression or translocation to the cell membrane may not occur.

### 3.2. The APLNR is a glycosylated protein that is susceptible to heat denaturation

**Figure 4.**
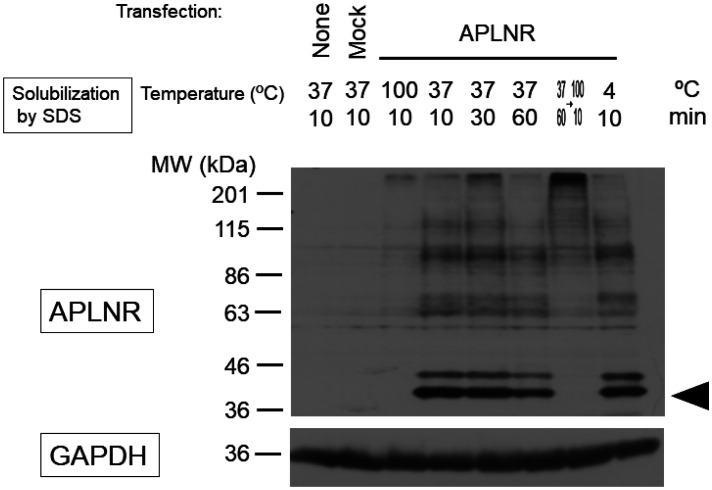
Solubilization of APLNR in HEK293TN cells. Cells were either cultured without any treatment (None) or transfected with a control (Mock) or APLNR expression plasmid, pcDNA3.1-APLNR (APLNR), for 3 days immediately after plating. Upon protein solubilization by SDS-PAGE, the cell lysates were incubated at 37 °C, 100 °C or 4 °C for 10, 30 or 60 min. GAPDH was used as an internal control. The black arrowhead indicates the desired molecular weight of the APLNR.

**Figure 5.**
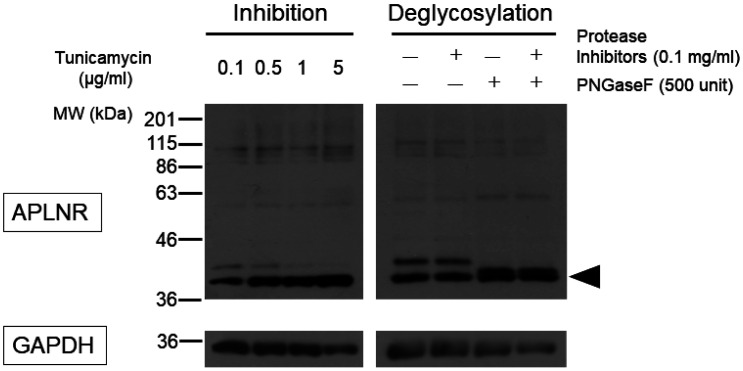
Inhibition of glycosylation and deglycosylation of APLNR. Left) After exposure to tunicamycin for 48 hr at indicated concentrations, proteins in HEK293TN cells expressing APLNR were solubilized at 37 °C for 10 min. Right) HEK293TN cells transfected with pcDNA3.1-APLNR were lysed and then reacted in the presence of proteolytic enzyme inhibitors or a de-N-glycosylating enzyme, PNGase F. Thereafter, the reaction solution was solubilized at 37 °C for 10 min. GAPDH was detected as an internal control. The arrowhead indicates the desired molecular weight of APLNR.

**Figure 6.**
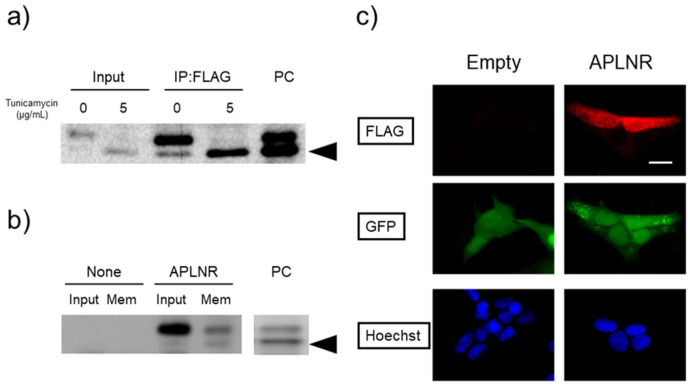
Cellular membrane localization of APLNR in HEK293TN cells. HEK293TN cells were transfected with the pCDH-GFP-APLNR vector. a) The cells were cultured with or without 5 µg/ml tunicamycin for 48 h. Lysates containing 100 µg of each protein were immunoprecipitated with an anti-FLAG antibody, solubilized at 37 °C for 10 min (IP: FLAG) and subjected to immunoblotting using an anti-APLNR antibody. b) Cells were incubated with cellular membrane-impermeant biotin to biotinylate membrane proteins, and a lysate containing 250 µg of biotinylated proteins was precipitated using Neutravidin-beads and solubilized at 37 °C for 10 min (Mem). a, b) The precipitates were subjected to immunoblotting using an anti-FLAG antibody. PC: positive control, cell lysate transfected with pcDNA3.1-APLNR. For the total lysate (input) or PC, 10 µg protein aliquots were used. b) Input and Mem of non-transfected HEK293TN cells were prepared as negative controls (none). c) Cells were fixed and subjected to immunostaining using a FLAG antibody to detect APLNR expression. Bar: 20 µm. A negative control experiment with an empty vector, pCDH-GFP, was also prepared (empty).

Using the standard sodium dodecyl sulfate-polyacrylamide gel electrophoresis (SDS-PAGE) method, we were unable to detect the APLNR protein after heating at 100 °C for 10 min (Fig. 4; third lane from the left). However, upon solubilization at a lower temperature of 37 °C or 4 °C, two bands of approximately 42.6 kDa appeared, corresponding to the desired molecular weight of the APLNR. After incubation at 37 °C for 60 min and 100 °C for 10 min, no bands were observed (Fig. 4). Receptor proteins with a high proportion of hydrophobic amino acid residues may form insoluble aggregates when heated during the SDS solubilization process, and lower temperatures can be more effective in such cases [22]. Apelin receptor may exhibit similar behavior. Treatment with tunicamycin or deglycosidase attenuated the signal intensity of the upper band, which exhibited slower mobility in electrophoresis. However, protease inhibitors did not affect the two bands (Fig. 5). To confirm its subcellular localization, the APLNR gene was subcloned into a plasmid co-expressing GFP. Similarly, in the subcloned plasmid, APLNR bands appeared after solubilization at 37 °C, and the upper band exhibited slightly slower migrating, which intensified and converted to a faster migrating band after tunicamycin treatment. Immunoprecipitation enhanced the intensity of APLNR bands (Fig. 6a). Furthermore, immunostaining revealed the predominant intracellular distribution of APLNR (Fig. 6c). Concentration of the upper band with slow mobility, detected in the biotinylated membrane proteins, using avidin beads resulted in decreased band intensity (Fig. 6b). These findings suggest that although the APLNR protein is overexpressed in cells, only a small amount of protein translocates to the plasma membrane.

### 3.3. Evidence suggests that the APLNR undergoes modifications in the mouse spinal cord

**Figure 7.**
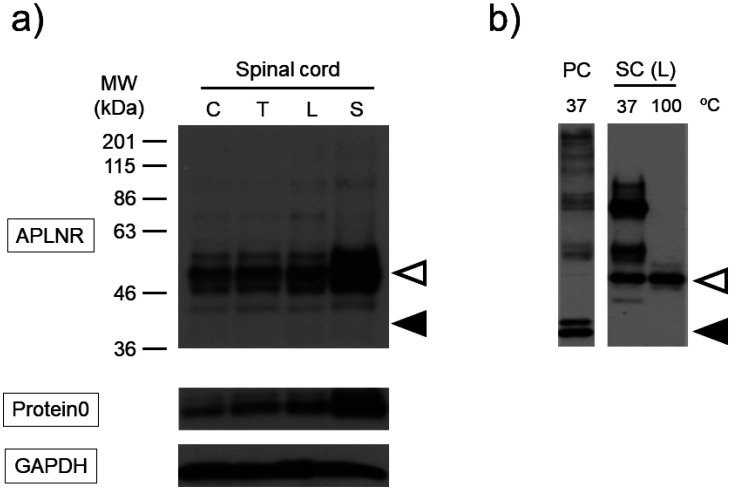
Property for APLNR in the mouse spinal cord. Representative immunoblot against APLNR and Protein0 in the spinal cord from three mice. a) Mouse cervical (C), thoracic (T), lumbar (L) and sacral (S) spinal cords were dissected, and the resulting proteins were solubilized at 100 °C and subjected to immunoblotting to detect APLNR, Protein0 and GAPDH. b) Mouse lumbar spinal cords were dissected, and proteins were solubilized at lower (37 °C) or normal (100 °C) temperatures. Lysate of HEK293TN transfected with pcDNA3.1-APLNR solubilized at 37 °C was used as a positive control (PC). White and black arrowheads indicate the main band in the spinal cord and the desired molecular weight of APLNR, respectively.

**Figure 8.**
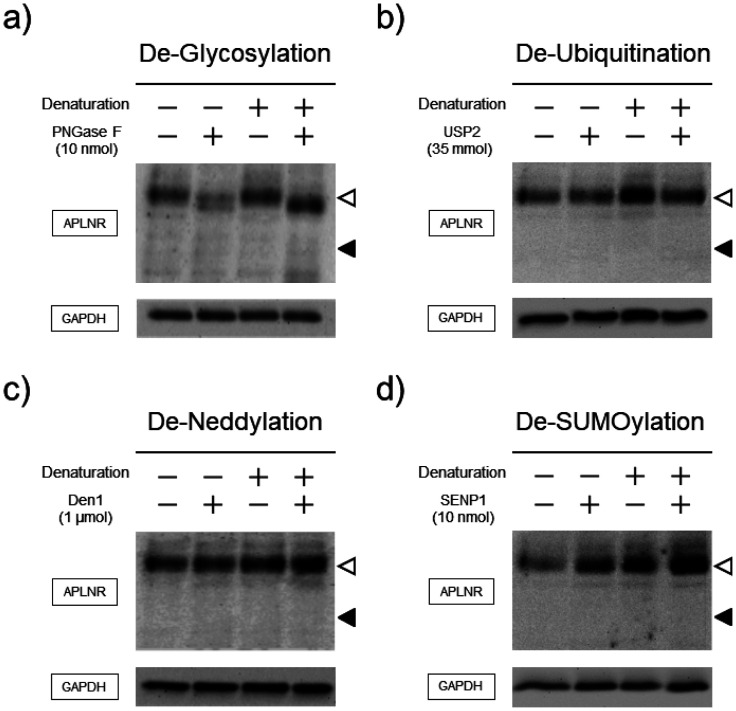
Possible isopeptidation of APLNR in the mouse spinal cord. Mouse spinal cords were dissected and lysed. Lysates were incubated either with or without denaturation and/or enzymes to introduce a) de-glycosylation by PNGaseF, b) de-ubiquitination by USP2, c) de-neddylation by Den1 or d) de-SUMOylation by SENP1 under appropriate enzyme conditions, as described in the Materials and Methods. After the reaction, APLNR and GAPDH were detected using immunoblotting. n = 3 biologically independent mice.

Immunoblot analysis showed the presence of bands corresponding to the molecular weight of APLNR and Protein0, markers of Schwann cells, in all mice spinal cord fractions, particularly in the sacral spinal cord fraction. The bands of the antibody against APLNR were approximately 10 kDa higher than the expected molecular weight (Fig. 7a). Solubilization of proteins from the lumbar spinal cord at a lower temperature of 37 °C resulted in the observation of multiple bands, but none of them corresponded to the desired molecular weight (Fig. 7b). In vitro reactions that cleave PTMs often require prior denaturation of the target protein. However, regardless of whether the protein was denatured or not, deglycosylation of APLNR occurred, causing a slight shift in the band migration towards the lower side with increased mobility. Notably, the deglycosylated band still appeared at a position higher than the expected molecular weight. However, regardless of the degeneration process, the de-ubiquitinating, de-NEDDylation or de-SUMOylation enzymes did not reveal any changes in the band patterns (Fig. 8).

## 4. Conclusions

The purpose of our study was to investigate and elucidate the intracellular signaling mechanisms of the APLNR. Previous studies have suggested that elucidating PTMs of the APLNR is necessary for this purpose. Although some receptors can be functionally characterized by expressing them in HEK293 cells [23], this was not the case for APLNR. Multiple studies have mentioned that the thermosensitive degradation of the APLNR needs to be addressed, but our study is the first to suggest that PMTs may be involved in resolving this issue. Previous studies have reported the instability of APLNR expressed using artificial methods such as gene transfection [24]. Wild-type APLNR has low protein yields and requires mutations for structural studies using X-ray crystallography [25]. This study showed that APLNR protein remains stable at or below 37 °C but denatured at higher temperatures, rendering it undetectable by antibodies (Fig. 4). Hydrophobic proteins such as membrane receptors, aggregate and hold when heated, but aggregation is suppressed at lower temperatures [22]. These differences may be attributed to PTMs. Furthermore, the expressed APLNR appeared to be inadequately localized to the cell membrane, as indicated by its higher concentration in the immunoprecipitate compared to the cell membrane fraction (Fig. 6). As proteins mature via pathways mediated by the endoplasmic reticulum and Golgi apparatus, insufficient PTMs may result in their accumulation in intracellular organelles. In the mouse spinal cord, APLNR was detected at a molecular weight 10 kDa higher than expected and was thermally stable, suggesting its conjugation with other proteins to gain non-denaturing properties (Fig. 7).

No APLNR isoforms have been reported in the GPCR database (GPCRdb), where GPCR isoforms can be searched (https://gpcrdb.org/protein/apj_human/). The additional 10 kDa molecular weight of APLNR could potentially result from a PTM involving covalent bonds; however, we did not identify the exact modifiers responsible. These modifications are important for improving thermostability and may also affect the functional expression of APLNR. Since ubiquitin is an 8.6 kDa protein, it is a likely candidate for causing the observed molecular weight shift of APLNR. However, since ubiquitinated proteins undergo further polyubiquitination and are degraded by the proteasome system, it is unlikely that polyubiquitination is the PTM of APLNR. SUMO and NEDD are modified at lysine residues by an E1–E3-mediated mechanism similar to ubiquitin; however, unlike ubiquitin, polymerization does not occur [26]. Unfortunately, our prediction did hold true, and neither SUMO nor NEDD was found to be associated with APLNR. Other proteins such as ATG8, ATG12, URM1, UFM1, FAT10 and ISG15 [27] have approximate molecular weights of 10 kDa and undergo isopeptidation. Although these modifications are uncommon, a comprehensive analysis of PTMs for APLNR is required. The glycosylphosphatidylinositol (GPI) anchor is a modification of approximately 10 kDa and is primarily associated with anchoring glycolipids to soluble proteins to lipid bilayer membranes; however, transmembrane proteins do not typically require the GPI anchor [28]. While acetylcholinesterase, alkaline phosphatase and CD90 are known to be modified with GPI anchors, the prediction by NEtGPI (https://services.healthtech.dtu.dk/services/NetGPI-1.1/) suggests that APLNR is not GPI-modified. Nevertheless, of the aforementioned three genes, only acetylcholinesterase is predicted to be GPI-modified, indicating the possibility of GPI modification on APLNR (data not shown). Although the specific PTM that confers stability to APLNR remains unknown, it is evident that APLNR was expressed in an unstable manner under the experimental conditions employed in this study.

In a previous study, we found that APLNR was expressed in motor neuron cell bodies in the mouse spinal cord [13]. In this study, immunoblotting revealed a substantial number of APLNR-positive bands in the sacral fraction, ranging from the cervical to sacral regions of the mouse spinal cord (Fig. 7). Compared with other spinal cord fractions, the sacral fraction contained peripheral nerve axons (cauda equina). Similarly, the lumbar spinal cord fraction exhibited strong APLNR-positive band intensity (data not shown). Schwann cells, responsible for myelinating peripheral nerve axons, express Gpr126, Gpr44 and LPA1, which are involved in their maturation, myelination and interaction with neuronal axons [29]. In particular, Gpr126 may promote the differentiation of Schwann cells by activating adenylate cyclase and increasing cAMP levels. In addition, Gpr126 is coupled with Gs and Gi proteins, which decreases cAMP concentration and activates Ras and PI3K cascades [29]. The expression of APLNR in Schwann cells suppresses their differentiation by decreasing cAMP levels. Gpr126 is also expressed in motor neuron axons, suggesting that Gpr126 signaling regulates cAMP in both Schwann cells and motor neurons. Therefore, APLNR may be expressed in Schwann cells and/or motor neurons and potentially at the nodes of Ranvier. The GABA_B_ receptor [30] and protease-activated receptor 1 (PAR-1) [31] are present on the nodes of Ranvier and are involved in myelination and conduction blockage during brain hemorrhage.

Apelin receptor exhibits diverse functions in various cell types, including those in the cardiovascular and nervous systems. Therefore, APLNR in HEK cells used in this study may not have a specific mechanism of action. We believe that activation of ERK may be due to activation of the autophagy pathway to degrade APLNR that cannot reach the cell membrane and accumulates within the cell. In fact, according to Nuria Martinez-Lopez et.al., activation of ERK by autophagosome formation has been reported [32]. (1) Since this is a reaction caused by the agonist expressed in the membrane, we believe that activation via β-arrestin does not contribute as the result that it did not reach the cell membrane this time, but it will reach the membrane in the future. We believe that it is necessary to proceed with analysis of the relationship with PTM. (2) The reagent used for this data was only (Pyr^1^)-apelin-13, but similar studies were conducted using ML221 as an APLNR agonist, and the results were the same, so the difference between isoforms is I concluded that it would not be possible, but I am thinking of considering isoforms next time. Other studies investigating APLNR in HEK cells have demonstrated the activation of signaling pathways [14]. Transfection experiments using vectors have shown a wide range of effective apelin concentrations. The expression pattern in HEK cells may change in vitro depending on the experimental conditions, such as the plasmid vector used. Therefore, it would be worthwhile to consider transfecting COS7 or CHO-K1 cells, which are commonly used for protein expression after gene transfer, as well as Neuro2A or NSC-34 cells as potential neuronal cell line candidates.

In vivo experiments have reported variations in the molecular weight of APLNR among different tissues. For example, 43 kDa in the mouse heart [33] and 50 kDa was observed in the spinal cord in this study, suggesting that PTMs differ between central and peripheral tissues. To elucidate the details of APLNR signal transduction, it is essential to establish an assay system that stably expresses APLNR on the cell membrane. To achieve this, we plan to reassess various experimental conditions and continue analyzing the physiological and pathophysiological functions mediated by APLNR.
